# Extracellular Signal-Regulated Kinase 1/2 Signaling Pathway Is Required for Endometrial Decidualization in Mice and Human

**DOI:** 10.1371/journal.pone.0075282

**Published:** 2013-09-24

**Authors:** Chae Hyun Lee, Tae Hoon Kim, Jae Hee Lee, Seo Jin Oh, Jung-Yoon Yoo, Hyo Suk Kwon, Young Im Kim, Susan D. Ferguson, Ji Yeon Ahn, Bon Jeong Ku, Asgerally T. Fazleabas, Jeong Mook Lim, Jae-Wook Jeong

**Affiliations:** 1 Department of Obstetrics, Gynecology & Reproductive Biology, Michigan State University College of Human Medicine, Grand Rapids, Michigan, United States of America; 2 Department of Agricultural Biotechnology, Seoul National University, Seoul, South Korea; 3 WCU Biomodulation Program, Seoul National University, Seoul, South Korea; 4 Cancer Research Institute, Seoul National University Hospital, Seoul, South Korea; 5 Department of Internal Medicine, Chungnam National University School of Medicine, Daejeon, South Korea; Baylor College of Medicine, United States of America

## Abstract

Decidualization is a crucial change required for successful embryo implantation and the maintenance of pregnancy. During this process, endometrial stromal cells differentiate into decidual cells in response to the ovarian steroid hormones of early pregnancy. Extracellular signal-regulated protein kinases 1 and 2 (ERK1/2) are known to regulate cell proliferation and apoptosis in multiple cell types, including uterine endometrial cells. Aberrant activation of ERK1/2 has recently been implicated in the pathological processes of endometriosis and endometrial cancer. However, the function of ERK1/2 signaling during implantation and decidualization is still unknown. To determine the role and regulation of ERK1/2 signaling during implantation and decidualization, we examine ERK1/2 signaling in the mouse uterus during early pregnancy using immunostaining and qPCR. Interestingly, levels of phospho-ERK1/2 were highest within decidual cells located at the implantation sites. Expression levels of ERK1/2 target genes were also significantly higher at implantation sites, when compared to either inter-implantation sites. To determine if ERK1/2 signaling is also important during human endometrial decidualization, we examined levels of phospho-ERK1/2 in cultured human endometrial stromal cells during *in vitro* decidualization. Following treatment with a well-established decidualization-inducing steroidogenic cocktail, levels of phospho-ERK1/2 were markedly increased. Treatment with the ERK1/2 inhibitor, U0126, significantly decreased the expression of the known decidualization marker genes, *IGFBP1* and *PRL* as well as inhibited the induction of known ERK1/2 target genes; *FOS*, *MSK1*, *STAT1*, and STAT3. Interestingly, the phosphorylation level of CCAAT/ enhancer binding protein β (C/EBPβ), a protein previously shown to be critical for decidualization, was significantly reduced in this model. These results suggest that ERK1/2 signaling is required for successful decidualization in mice as well as human endometrial stromal cells and implicates C/EBPβ as a downstream target of ERK1/2.

## Introduction

Embryo implantation is a key for the successful establishment of pregnancy and requires crosstalk between the embryo and the receptive uterus. During early pregnancy, the endometrium undergoes cyclic changes in response to the ovarian steroid hormones, progesterone (P4) and estrogen (E2) [1]. In response to signals released by the newly fertilized embryo, uterine stromal cells proliferate and transform from spindle-shaped fibroblastic cells into large, round and multinucleated decidual cells in a process known as decidualization [2]. Upon transformation, decidualized cells acquire a secretory epithelioid-like phenotype characterized by cytoplasmic accumulation of glycogen and lipid droplets, as well as expansion of both the golgi complex and rough endoplasmic reticulum [3]. From a functional perspective, these cellular changes enable the production of various hormones, growth factors, and cytokines needed to promote uterine angiogenesis and hemostasis during trophoblast invasion and placenta formation [3]. The decidualization process also enables establishment of maternal immunological tolerance to fetal antigens and protects the conceptus from environmental insults [4,5]. Therefore, perturbations in the decidualization process generally result in either implantation failure or placental dysfunction.

It is known that this differentiation event is linked to morphological, biochemical and genetic changes triggered by the receptors for the ovarian steroids, estrogen and progesterone. Previous research focused on identifying genes which are differentially expressed in “pre-decidualized” versus “decidualized” state have contributed to our understanding of this process [6]. However, our knowledge of the molecular and functional mechanisms controlling this process and its potential associations with implantation and decidualization defects is still far from complete.

Abnormal decidualization of endometrial stromal cells has been correlated with unexplained infertility, miscarriage and endometrial pathologies such as endometriosis [7,8,9,10]. It has been reported that endometriosis patients have a reduced decidualization capacity. Additionally, many studies have demonstrated a potential link between extracellular signal-regulated kinase 1/2 (ERK1/2) signaling and endometriosis [11,12,13]. Specifically, ERK1/2 has been shown to influence cAMP-dependent cell cycle regulation in cultured human endometrial stromal cells (hESCs) and phospho-ERK1/2 is aberrantly increased in hESCs derived from women with endometriosis [7,12]. Additional studies have directly linked the enhanced proliferation and survival of hESCs derived from women with endometriosis (as compared with healthy controls) with abnormal activation of the ERK1/2 signaling pathways [13]. However, the function of ERK1/2 signaling during decidualization and how this may be altered in women with endometriosis is not known.

ERK1/2 is a member of the well-known MAPK pathway and implicated in the regulation of cellular proliferation and differentiation in multiple organ systems. ERK1/2 is activated by the upstream Ras/Raf/MEK signaling cascade via phosphorylation on residues threonine 202 and tyrosine 204. This activated form of ERK1/2 (phospho-ERK1/2) can subsequently phosphorylate various transcription factors to regulate cellular proliferation, differentiation, apoptosis and inflammation [14]. It has also been reported that EGF regulates the growth and differentiation of cultured human endometrial stromal cells [15]. Based on the above evidence, we hypothesize that ERK1/2 signaling, which is regulated by EGFR, may be one critical pathway for endometrial stromal cell decidualization.

In the present study, we investigated the role of ERK1/2 during decidualization in the mouse uterus as well as in cultured human primary endometrial stromal cells. We observed that activation of ERK1/2 signaling coincided with the onset of decidualization in both mice and human. Inhibition of ERK1/2 phosphorylation by U0126, a specific MEK inhibitor, significantly decreased this decidualization process. Our results indicate that the regulation of ERK1/2 activity is required during the endometrial stromal cell decidualization process.

## Materials and Methods

### Animals and tissue collection

Animals were maintained in a designated animal care facility according to the Michigan State University’s Institutional Guidelines for the care and use of laboratory animals. All animal procedures were approved by the Institutional Animal Care and Use Committee of Michigan State University (12/10-198-00). For early pregnancy studies, wild-type female C57BL/6 mice at 8 weeks of age were mated with wild-type male mice. The morning a vaginal plug observed was designated 0.5 days post-coitum (dpc). Uteri samples were collected from 0.5 dpc to 7.5 dpc of pregnancy, and separated into implantation site (IS) and inter-implantation site (IIS) on 5.5 dpc and 7.5 (n = 3). Artificial decidualization studies were performed as previously described [16]. In brief, ovariectomized mice were injected with 100ng of Estradiol (E2) daily for 3 days. After two days rest, daily injections of Progesterone (P4, 1 mg/mouse) and E2 (6.7 ng/mouse) followed for three days. Six hours after the third injection of P4 plus E2, the left uterine horn was mechanically stimulated by scratching the full length of the anti-mesometrial side with a burred needle, while the right horn was left unstimulated as a control. Daily injections of P4 (1 mg/mouse) and E2 (6.7 ng/mouse) were then continued for five more days. The mice were sacrificed by cervical dislocation after placing the mice under anesthesia, Avertin (2,2,-tribromoethyl alcohol, Sigma–Aldrich, St. Louis, MO). Uterine samples were collected on day 1 and day 5. Uterine tissues were flash frozen at the time of dissection and stored at -80°C.

### Immunohistochemistry

Uterine samples were fixed in 4% paraformaldehyde and paraffin-embedded as previously described [17]. Uterine cross sections from paraffin-embedded tissue were cut into 5µm sections, mounted on silane-coated slides, deparaffinized and rehydrated in a graded alcohol series. Sections were pre-incubated with 10% normal serum in phosphate-buffered saline (PBS-pH 7.5) and then incubated overnight with either anti-ERK1/2 (Cell Signaling, Danvers, MA), or anti-phospho-ERK1/2 (Cell Signaling, Danvers, MA) antibodies in PBS supplemented with 10% normal goat serum. The next day, sections were washed with PBS and incubated with a secondary antibody conjugated to horseradish peroxidase (Vector Laboratories, Burlingame, CA) for 1 hour at room temperature and immunoreactivity detected using the Vectastain elite ABC kit according to manufacturer’s instructions (Vector Laboratories, Burlingame, CA). In this method, a diaminobenzamine substrate is applied and immunoreactivity visualized as a brown precipitate.

### Primary human endometrial stromal cell cultures and *in vitro* decidualization

Human primary endometrial stromal cells (hESCs) were obtained from The Michigan State University’s Center for Women’s Health Research Female Reproductive Tract Biorepository with MSU Biological Institutional Review Board approval. All samples were obtained with written informed consent from pre-menopausal women undergoing hysterectomy for benign indications (prolapse and uterine fibroids) who had not been on any hormonal therapies for a minimum of 90 days prior to surgery. Diagnosis and endometrial dating of all samples was confirmed via pathological analysis. Primary endometrial stromal cells were isolated from pure endometrial tissue by collagenase digestion as previously described [18]. In brief, endometrial tissue was minced and digested twice at 37°C in buffered saline supplemented with 0.5% collagenase (Sigma-Aldrich, St. Louis, MO) and 0.002% DNase (Sigma-Aldrich, St. Louis, MO) prior to filtration through 70µm sterile sieves to exclude epithelium. The remaining hESCs were allowed to attach on cell culture treated flasks and grown in phenol red–free RPMI-1640 medium (Gibco, Grand Island, NY) containing 0.1 mM sodium pyruvate (Gibco, Grand Island, NY), 10% fetal bovine serum (FBS; Gibco, Grand Island, NY) depleted of steroids by pre-treatment with dextran-coated charcoal (Sigma Aldrich, St. Louis, MO) (Charcoal-stripped FBS; CS-FBS), and 1% penicillin streptomycin (P/S; Gibco, Grand Island, NY). To induce *in vitro* decidualization, cells were washed with PBS and transferred to OPTI-MEM medium (Gibco, Grand Island, NY) containing 2% CS-FBS, 10nM estradiol (E2, Sigma-Aldrich, St. Louis, MO), 1mM medroxyprogesterone acetate (MPA; Sigma-Aldrich, St. Louis, MO), 50 µM cAMP (Sigma-Aldrich, St. Louis, MO), and 1% P/S. Differentiation medium was changed every 48 hours and treatment lasted for 6 days. For MEK inhibitor treatment, 10 µM of U0126 (Cell Signaling, Danvers, MA) was added to the differentiation medium on day 3 of differentiation. All results from hESCs *in vitro* studies were confirmed using primary human endometrial stromal cells obtained from at least three independent biological replicates (n=3).

### Western blot analysis

Cellular proteins were extracted using lysis buffer (1M Tris, 0.5M EDTA, 5M NaCl, 10% NP40) in distilled water supplemented with both a protease inhibitor cocktail (Roche, Indianapolis, IN) and a phosphatase inhibitor cocktail (Sigma Aldrich, St. Louis, MO). Ten µg of protein lysates were electrophoresed using SDS-PAGE and transferred onto polyvinylidene difluoride membrane (Millipore Corp., Bedford, MA, USA). Casein (0.5% v/v) was used to block the membrane prior to exposure to anti-ERK1/2 (Cell Signaling, Danvers, MA), anti-phospho-ERK1/2 (Cell Signaling, Danvers, MA), anti-C/EBPβ (Santa Cruz Biotechnology, Santa Cruz, CA), anti-phospho-C/EBPβ (Cell Signaling, Danvers, MA) or anti-Actin (Santa Cruz Biotechnology, Santa Cruz, CA) antibody immunoblotting. Immunoreactivity was visualized by incubation with a horseradish peroxidase-linked secondary antibody followed by exposure to ECL reagents according to manufacturer’s instructions (GE Healthcare Biosciences, Piscataway, NJ).

### Immunofluorescence staining

Uterine sections from paraffin-embedded tissue were cut at 6 µm and mounted on silane-coated slides, deparafinized and rehydrated in a graded alcohol series. After further washing, slides were exposed to anti-phospho-ERK1/2 (1:200) and anti-Ki67 (1:500; BD Bioscience, San Diego, CA) antibodies overnight at 4°C and secondary antibodies for 1 hour at room temperature. Slides were counterstained by Vectashield mounting media with DAPI (Vector Laboratories, Burlingame, CA, USA).

hESCs were grown on glass coverslips to 90% confluency and subjected to decidualization treatment as described above. Upon completion of treatment, coverslips were washed with PBS, fixed with 4% paraformaldehyde for 30min at room temperature and permeabilized with 0.1% of Triton X-100 (Sigma-Aldrich, St. Louis, MO). After further washing, hESCs were exposed to primary antibodies overnight at 4°C and secondary antibodies for 1 hour at room temperature. Washed coverslips were then mounted onto microscope slides with a DAPI-impregnated mounting media (Vector Laboratories, Burlingame, CA) to enable nuclear visualization and images captured with a fluorescent microscope (Nikon Instruments Inc., Melville, NY) using software from NIS Elements, Inc. (Nikon, Melville, NY).

### RNA isolation and quantitative real-time PCR

Total RNAs were isolated using RNeasy total RNA isolation kit (Qiagen, Valencia, CA) according to manufacturer’s instruction. As a template for quantitative PCR, cDNAs were synthesized using quantitative PCR random hexamers and MMLV Reverse Transcriptase (Invitrogen Corp., Carlsbad, CA). Expression levels of *GFBP1, PRL, FOS, STAT1, STAT3*,*MSK1* in hESCs and *Serpine1, Fos, Elk1, cMyc, Stat1, Ubtf* and *Msk1* in early pregnant mouse uterus were measured by quantitative real-time PCR (Applied Biosystems, Foster City, CA) using ABI pre-verified or sequence-specific primer-probe sets, which were designed using Primer3 software (Whitehead Institute/MIT, Center for Genome Research, Cambridge, MA) (Table S1). Real-time PCR results were normalized against the housekeeping genes, 18S RNA.

### Statistical Analysis

Statistical analyses were performed using one-way ANOVA analysis followed by Tukey’s post hoc multiple range test or Student’s t-tests using the Instat package from GraphPad (San Diego, CA). *p* < 0.05 was considered statistically significant.

## Results

### ERK1/2 expression during early pregnancy in mice

**Figure 1:**
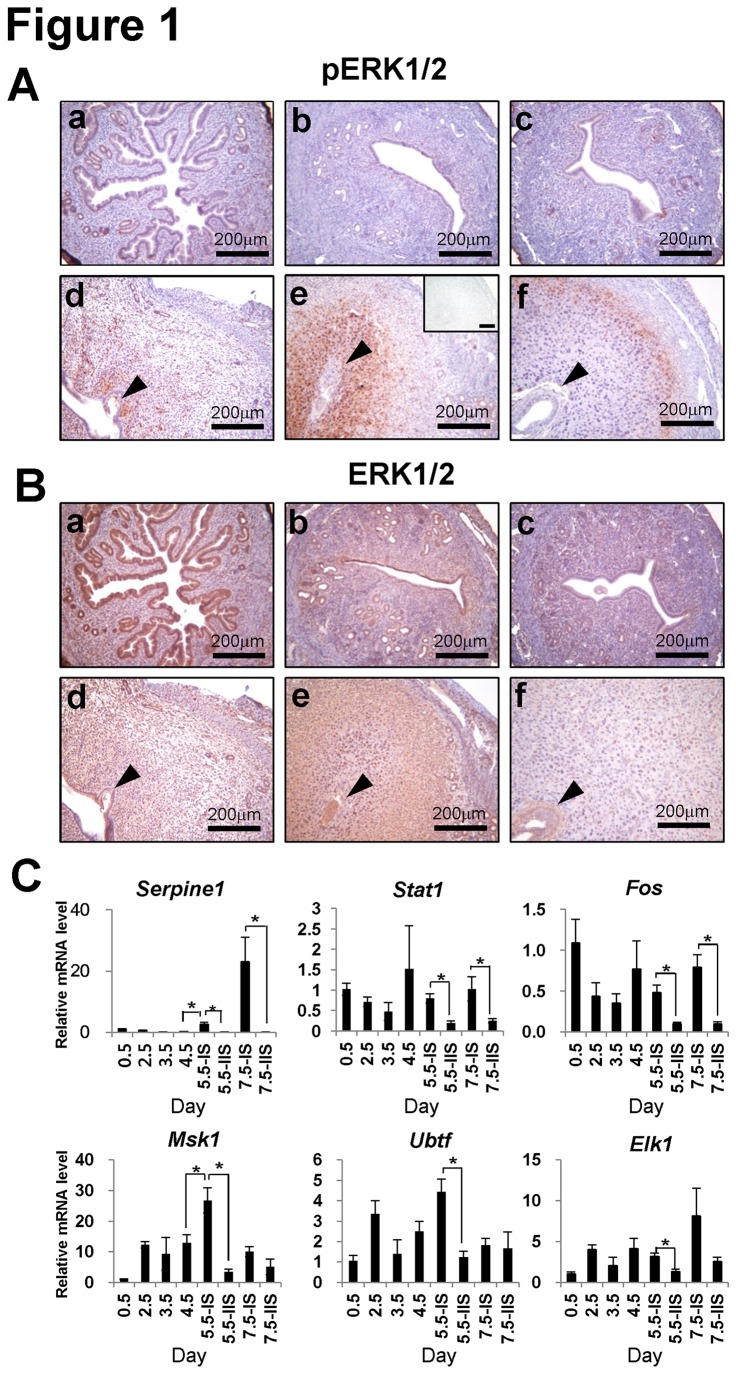
Expression of phospho-ERK1/2, total ERK1/2 and its target genes during early pregnant mice. (A and B) The localization pattern of phospho-ERK1/2 (A) and total ERK1/2 (B) were examined by immunohistochemistry in uteri of C57BL/6 mice on 0.5 (a), 2.5 (b), 3.5 (c), 4.5 (d), 5.5 (e) and 7.5 (f) dpc. Normal rabbit IgG was used as a negative control (e, inset). (C) Expression of ERK1/2 target genes during early pregnancy mice. The expression of *Serpine1, Stat1, Fos, Ubtf, Msk1* and *Elk1* were examined in uteri of C57BL/6 mice on 0.5-7.5 dpc. The results represent the mean ± SE. *, *p*<0.05; IS: implantation sites, IIS: inter-implantation sites. Scale bar: 200 µm.

To investigate the expression of ERK1/2 during early pregnancy, levels of total and phosphorylated ERK1/2 were examined in uteri of C57BL/6 female mice during early pregnancy (Figure 1). Immunohistochemical analysis of uterine cross sections revealed that expression of phospho-ERK1/2 was not detectable until 3.5 dpc. Interestingly, strong expression of phospho-ERK1/2 was present in sub-epithelial stromal cells at 4.5 dpc, extended into the primary decidual zone at 5.5dpc, and was next observed in the secondary decidual zone at 7.5 dpc (Figure 1A). However, total ERK1/2 was constitutively expressed throughout the entire uterus (Figure 1B). These results suggest that phosphorylation of ERK1/2 may play important role in decidualization during early pregnancy.

ERK1/2 is activated via dual phosphorylation on specific tyrosine (Tyr204) and threonine (Thr202) residues by mitogen-activated or extracellular signal-regulated protein kinase kinase (MEK) [14]. To determine if ERK1/2 signaling is activated at implantation sites, we examined the expression of known ERK1/2 target genes in the mouse uterus during early pregnancy. The expression of Serpin Peptidase Inhibitor, Clade E member 1 (*Serpine1*), Signal Transducer and Activator of Transcription 1 (*Stat1*), FBJ osteosarcoma oncogene (*Fos*), Upstream binding transcription factor (Ubtf), Mitogen- and Stress-activated protein kinase1 (*Msk1*), and a member of the ETS oncogene family (*Elk1*) were all significantly increased within the implantation site (IS) as compared to the inter-implantation site (IIS) at 5.5 dpc. The levels of *Serpine1, Stat1* and *Fos* were also significantly increased within the IS at 7.5 dpc as compared with IIS (Figure 1C). These results suggest specific activation of ERK1/2 signaling at the IS occurs in a spatial-temporal manner.

**Figure 2:**
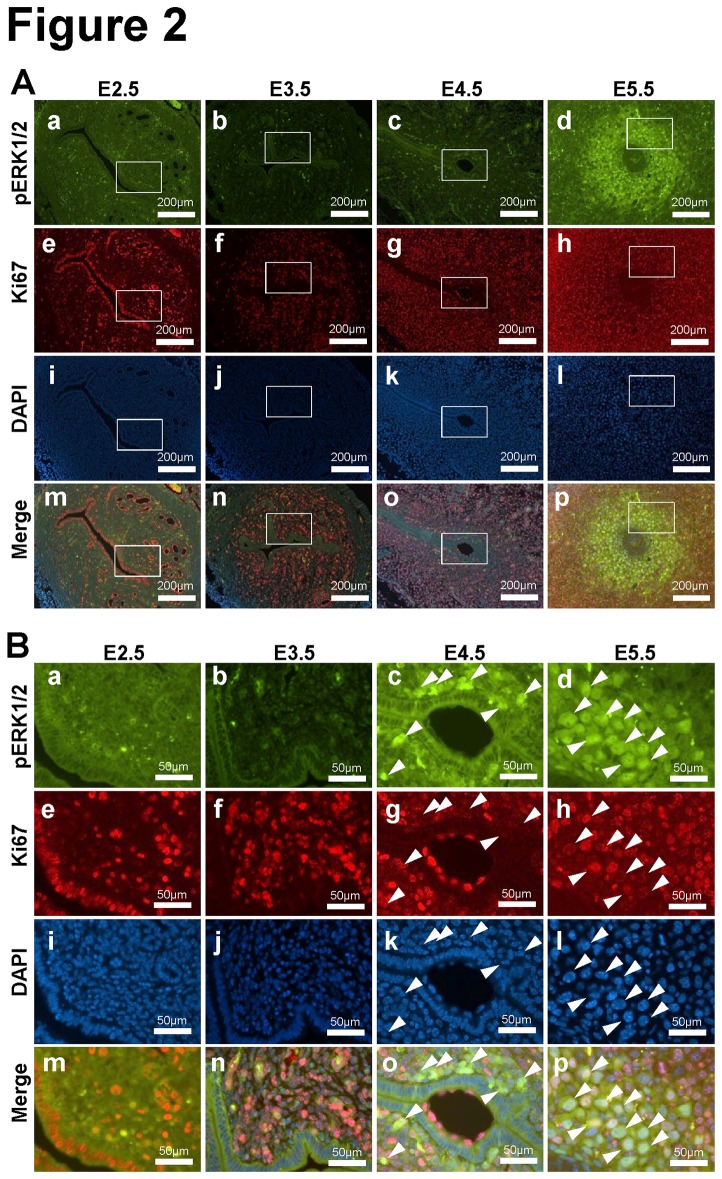
Relationship between phospho-ERK1/2 and proliferation during early pregnancy. (A) Immunofluorescence analysis of phospho-ERK1/2 (green; a, b, c and d) and Ki67 (red: e, f, g and h) was performed in uteri of C57BL/6 mice on 2.5 (a, e, i and m), 3.5 (b, f, j and n), 4.5 (c, g, k and o) and 5.5 (d, h, l and p) dpc. Images (m, n, o and p) were merged with DAPI staining (i, j, k and l). (B) Higher magnification images of the insets. Arrowheads indicates positive phospho-ERK1/2 cells.

The uterus undergoes dynamic changes during pregnancy in mice. Uterine receptivity requires a dialogue between the hormonally primed maternal endometrium and the free-floating blastocyst. Endometrial stromal cells proliferate, avert apoptosis, and undergo decidualization in preparation for implantation. To determine if phosphorylation of ERK1/2 is associated with this stromal proliferation during early pregnancy, we performed double immunofluorescence for phospho-ERK1/2 and Ki67, a proliferation marker (Figure 2). Abundant expression of Ki67 was detected in epithelial cells and some stromal cells at 2.5 dpc. Proliferation markedly increased within the stromal, but not epithelial compartment at 3.5 dpc, as determined by Ki67 immunostaining. On 4.5 dpc, a time associated with initiation of attachment, endometrial stromal cells surrounding the blastocyst undergo decidual transformation. The expression of phospho-ERK1/2 was strongly detected at decidual cells near embryo on 4.5 dpc. However, phospho-ERK1/2 positive cells did not express Ki67. The expression of phospho-ERK1/2 was extended into the primary decidual zone at 5.5 dpc. Again, on 5.5 dpc, Ki67 positive cells continued to be presented, but only a small numbers of cells co-expressed both Ki67 and phospho-ERK1/2. H-scoring/Image J analysis, revealed that, among phospho-ERK1/2 positive cells, 14.97% of cells were both Ki67 and phospho-ERK1/2 positive. The remaining 84.49% cells were phospho-ERK1/2 positive but Ki67 negative. The remaining phospho-ERK1/2 negative cells were highly active proliferative within non-decidual region. These results indicated that endometrial stromal cells begin to proliferate prior to implantation, whereas phospho-ERK1/2 expression is initiated within stromal cells near the embryo at the time of implantation. Our results suggest that phospho-ERK1/2 expression correlates with decidual differentiation.

**Figure 3:**
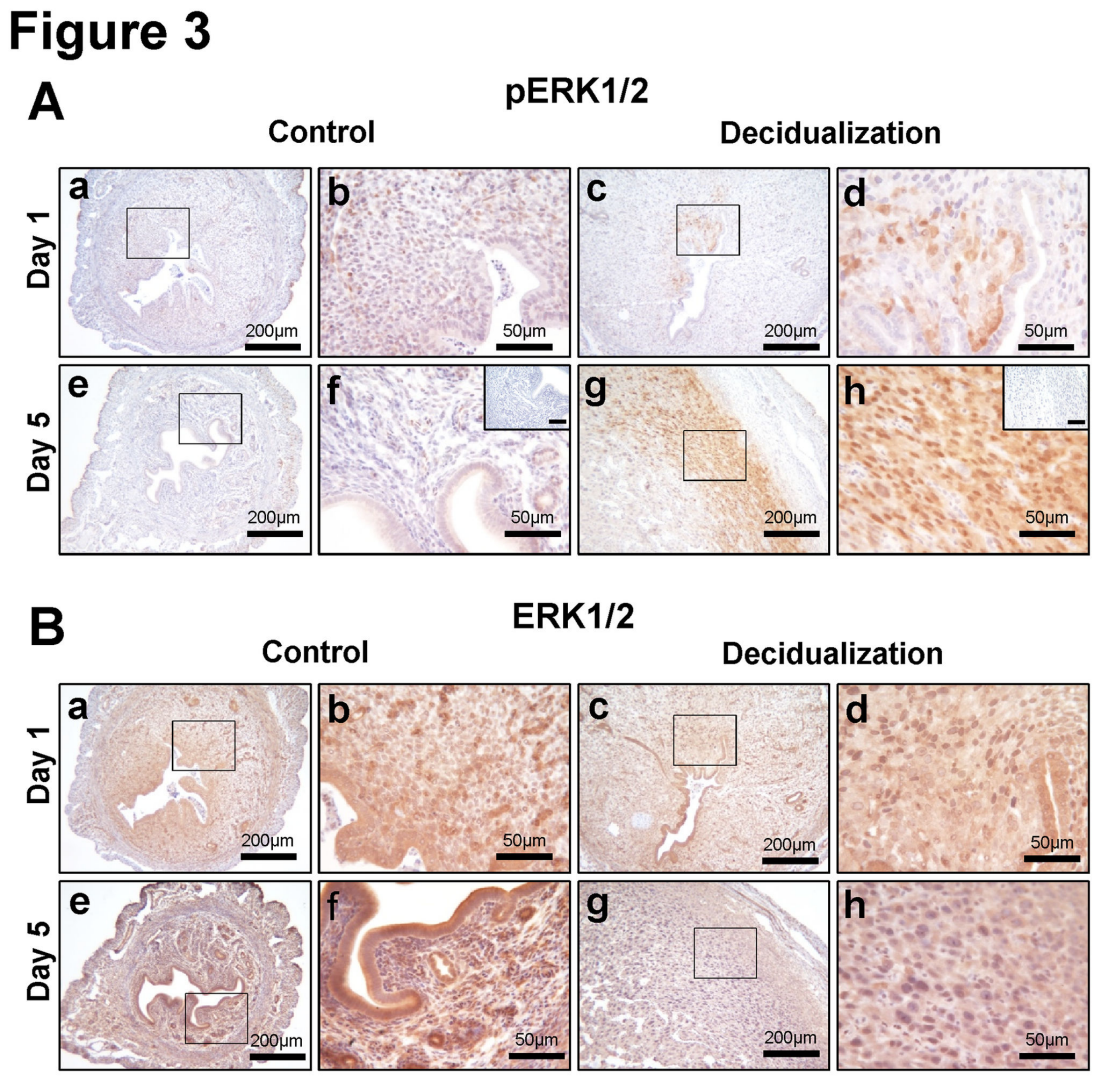
Expression of phospho-ERK1/2 and total ERK1/2 in artificially-induced decidual uterus of mice. Expression pattern of phospho-ERK1/2 (A) and total ERK1/2 (B) were investigated by immunohistochemistry after artificially-induced decidualization on day 1 and day 5. Expression of phospho-ERK1/2 was observed in stimulated horn but not in control horn. Total ERK1/2 was constitutively expressed both control and stimulated uteri on day 1 and day 5. Normal rabbit IgG was used as a negative control (A f and h, inset). Scale bar: 50 µm (inset).

The stromal cells which surround the embryonic implantation site undergo a differentiation process known as decidualization, which is essential for the establishment of a successful pregnancy. To examine whether activation of ERK1/2 signaling occurs during decidualization, ovariectomized C57BL/6 mice were hormonally primed and mechanically stimulated to mimic embryo implantation and induce artificial decidualization (see Materials and Methods). Phospho-ERK1/2 was detected only in decidual cells on day 1 and was strongly detectable on day 5 (Figure 3A). However, total ERK1/2 was consistently expressed in epithelium, stroma and decidua of control and decidualized uterine horns (Figure 3B). These results suggest that ERK1/2 proteins are activated by phosphorylation during decidualization process.

### ERK1/2 Expression in Human Endometrial Stromal Cells (hESCs) during In Vitro Decidualization

**Figure 4:**
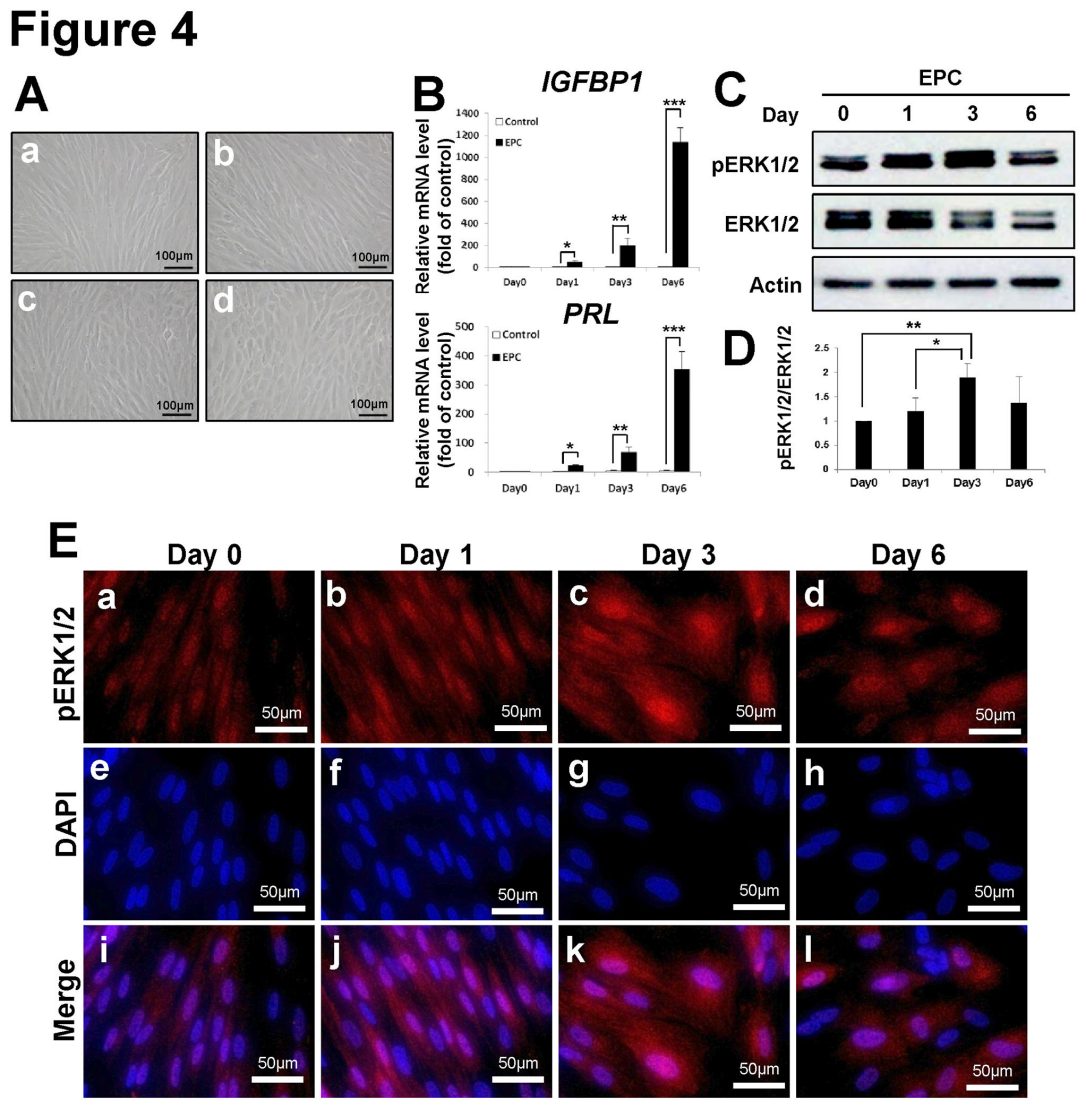
Expression of ERK1/2 during *in vitro* decidualization of human endometrial stromal cells (hESCs). (A) Morphological change of hESCs was observed during *in* *vitro* decidualization on day 0 (a), day 1 (b), day 3 (c) and day 6 (d). (B) Expression of decidualization marker genes, *IGFPB1* and PRL, was examined during *in* *vitro* decidualization. (C) Level of total ERK1/2 and phospho-ERK1/2 proteins was measured by western blot. (D) Quantification of pERK1/2 protein levels during *in* *vitro* decidualization. The results represent the mean ± SE. *, *p*<0.05; **, *p*<0.01; ***, p<0.001. (E) Increased expression of phospho-ERK1/2 (a, b, c and d) was detected on day 3 by immunofluorescence during *in* *vitro* decidualization. Images (i, j, k and l) were merged with DAPI staining (e, f, g and h).

To examine whether ERK1/2 is activated during human endometrial stromal cell differentiation, we used a well characterized *in vitro* model [18] to induce decidualization in cultured human primary endometrial stromal cells (hESCs) by treating the cells with estrogen, progesterone and cAMP. Prior to treatment, hESCs possessed a fibroblast-like morphology. After *in vitro* decidualization treatment, hESCs enlarged and became round in shape, typical of the decidual transformation (Figure 4A). Quantitative PCR analysis revealed significantly increased expression levels of the decidualization marker genes (*IGFBP1*and *PRL*) after treatment (Figure 4B). To investigate phospho-ERK1/2 levels during this process, western blot analysis was conducted on protein lysates from control hESCs or hormone-treated hESCs on day 0, 1, 3 and 6 of *in vitro* decidualization. The results show increased expression of phospho-ERK1/2 on the first 3 days of treatment which then tapered slightly on day 6 (Figure 4C and D). The level of phospho-ERK1/2 at day 3 was significantly increased compared to both day 0 and day 1 (189 ± 29% and 158 ± 24%, respectively). A marked decrease in phospho-ERK1/2 was evident between days 3 and 6; however this difference did not reach statistical significance. ERK1/2 is primarily located in the cytoplasm of resting cells, although overexpression of ERK1/2 can result in both cytoplasmic and nuclear localization [19]. Upon activation, ERK1/2 translocates from the cytoplasm to the nucleus, where it binds to and transcriptionally activates downstream target genes [19,20]. Therefore, we examined localization of total and phospho-ERK1/2 throughout decidualization. Immunofluorescent staining of both control and artificially decidualized hESCs showed that total ERK1/2 was consistently expressed in decidualized as well as non-decidualized cells (Figure S1). However, strong nuclear localization of phospho-ERK1/2 was detected on day 3 compared to day 0 and day 1 during *in vitro* decidualization (Figure 4E). These results indicate that nuclear accumulation of phospho-ERK1/2 is induced during *in vitro* decidualization of hESCs.

### Impact of pERK1/2 inhibition during in vitro decidualization in hESCs

**Figure 5:**
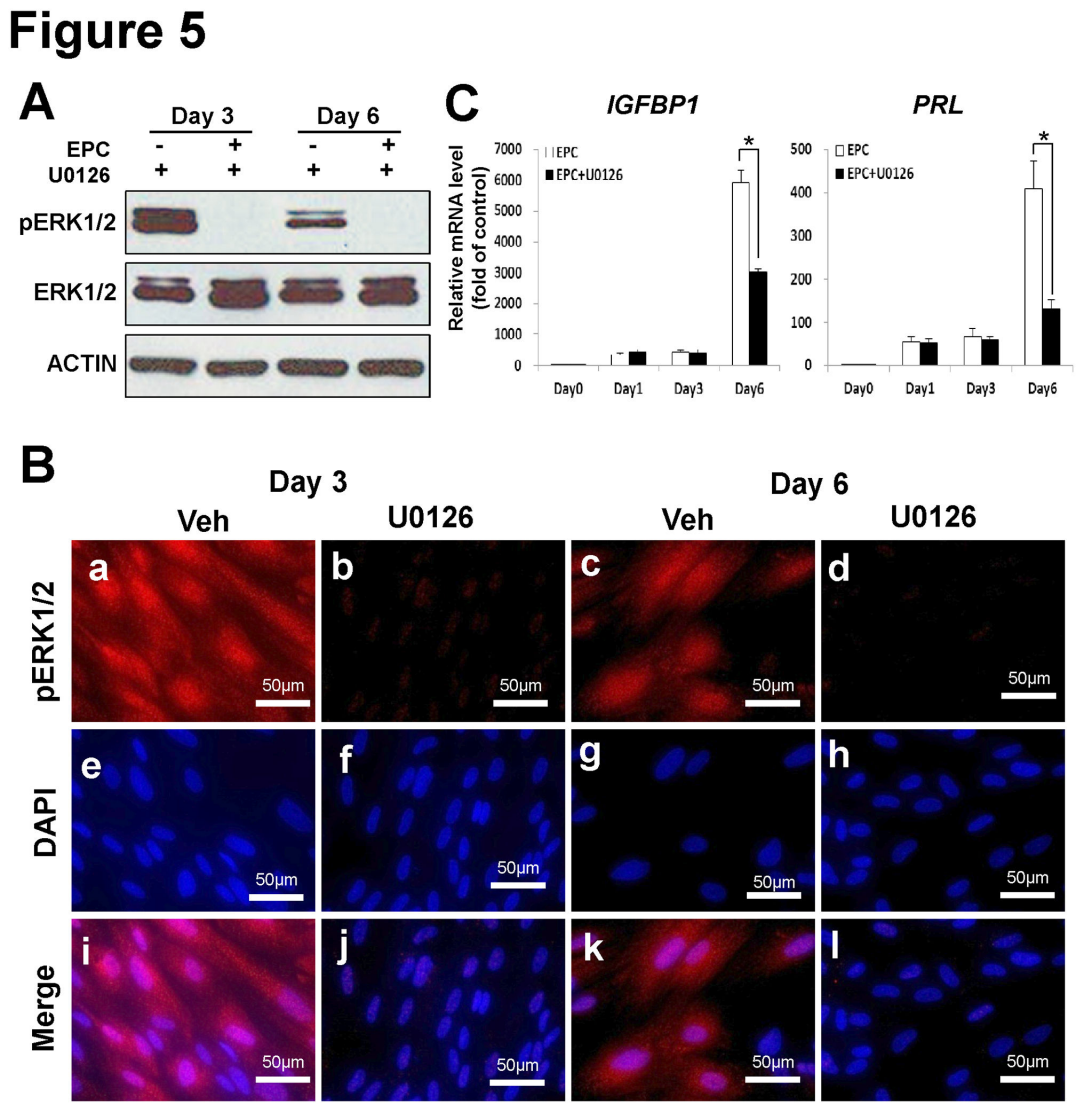
Effect of inhibition of ERK1/2 phosphorylation during *in vitro* decidualization of hESCs. ERK1/2 phosphorylation was inhibited by treatment with U0126 on day 3 of *in* *vitro* decidualization. (A and B) Inhibition of ERK1/2 phosphorylation (a, b, c and d) was confirmed by western blot (A) and immunofluorescence staining (B). Images (i, j, k and l) were merged with DAPI staining (e, f, g and h). (C) The expression of decidualization marker genes, *IGFBP1* and *PRL*, was significantly decreased after treatment with U0126. *, *p*<0.05.

To examine whether activation of ERK1/2 is required for human endometrial stromal cell decidualization, we inhibited phosphorylation of ERK1/2 using a known MEK inhibitor, U0126, during *in vitro* decidualization of hESCs. Successful inhibition of ERK1/2 phosphorylation was confirmed by western blot analysis and immunofluorescence staining. Results showed that total ERK1/2 levels were unchanged, whereas phosphorylation levels of ERK1/2 were significantly reduced in decidualized stromal cells treated with U0126 (Figure 5A). Nuclear accumulation of phospho-ERK1/2 was not detected in the inhibitor-treated cells on either day 3 or day 6 (Figure 5B). To determine whether ERK1/2 phosphorylation is required for decidualization, the expression of decidual marker genes, *IGFBP1* and *PRL* was examined. After treatment of inhibitor, the expression of *IGFBP1* and *PRL* were significantly reduced on day 6 in decidualized cells as compared with the vehicle-treated controls (Figure 5C). These results suggest that activation of ERK1/2 is required to induce decidualization in hESCs.

### The expression of ERK1/2 target genes following inhibition of ERK1/2 phosphorylation during in vitro decidualization

**Figure 6:**
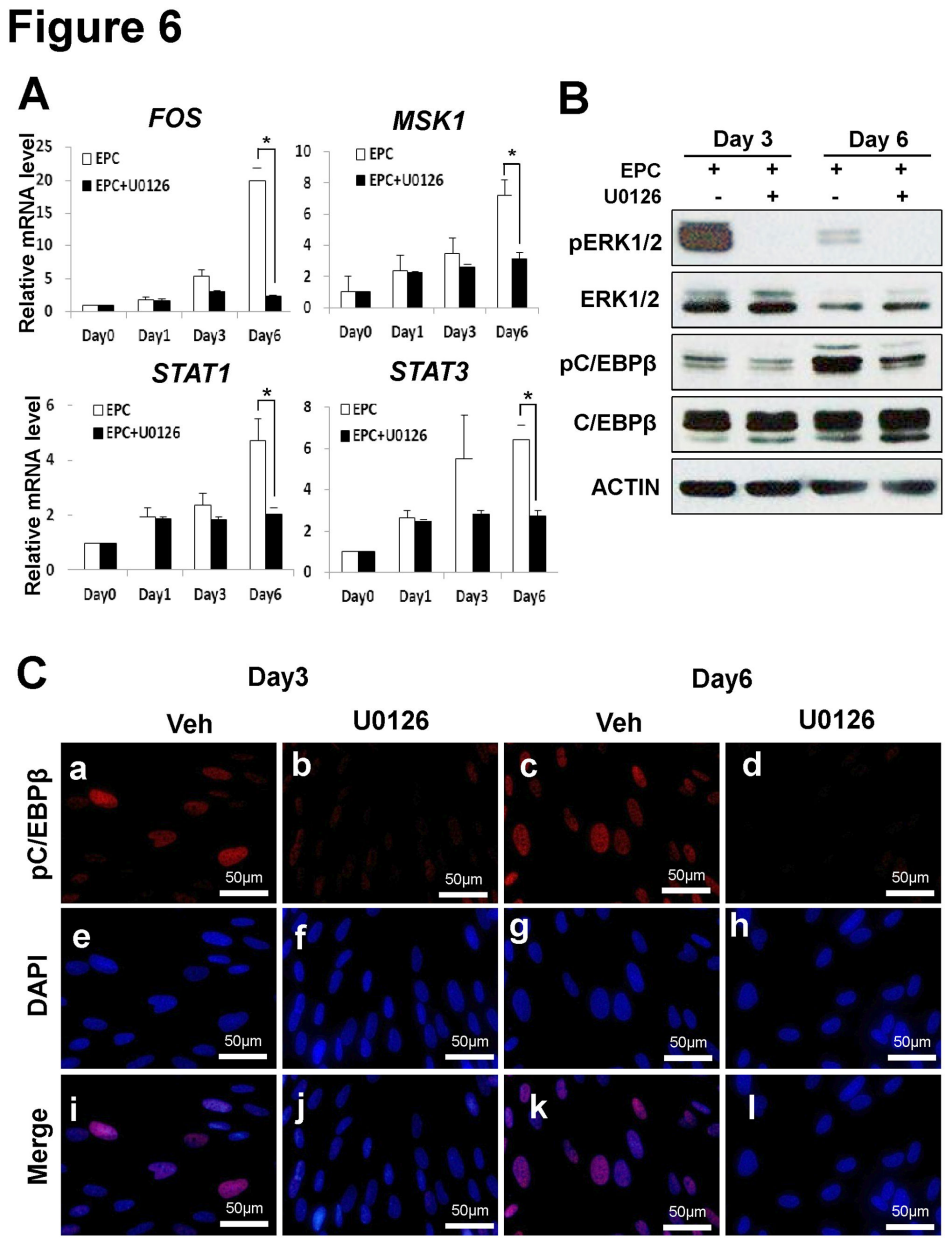
The levels of ERK1/2 targets were decreased by U0126 treatment during hESCs decidualization. (A) The expression of ERK1/2 target genes, *FOS, MSK1, STAT1* and *STAT3* was significantly decreased by inhibition of ERK1/2 phosphorylation during *in* *vitro* decidualization. *, *p*<0.05. (B and C) Phosphorylation of C/EBPβ was evaluated by western blot (B) and immunofluorescence staining (C). The levels of phospho-C/EBPβ (a, b, c and d) were reduced in hESCs treated with U0126 during decidualization. Images (i, j, k and l) were merged with DAPI staining (e, f, g and h).

To determine if downstream target genes of ERK1/2 are altered by inhibition of ERK1/2 phosphorylation during *in vitro* decidualization of hESCs, we examined the expression profiles of the following ERK1/2 target genes, *FOS, MSK1, STAT1*and *STAT3* by qPCR. As shown in Figure 6A, levels of *FOS, MSK1, STAT1* and *STAT3* were significantly reduced in decidualized hESCs treated with U0126. CCAAT/enhancer binding protein β (C/EBPβ) is a novel mediator of the biological actions of E2 and P4 in the uterus during early pregnancy [21,22]. C/EBPβ is required for decidualization in mice [21,22], non-human primates [23] and humans [24,25]. It is a critical regulator of hESCs proliferation and differentiation [24,26] and a known substrate of ERK1/2 [27,28]. To determine whether C/EBPβ expression is altered by inhibition of ERK1/2 phosphorylation during decidualization, we examined levels of total and phospho-C/EBPβ in decidualized hESCs in the presence or absence of U0126. Western blot analysis showed that phospho-C/EBPβ levels increased during decidualization on day 3 and day 6 but its levels were reduced in lysates from hESCs treated with U0126 as compared to vehicle-treated control (Figure 6B). The results of immunofluorescence staining showed that levels of nuclear phospho-C/EBPβ expression were reduced in decidualized hESCs treated with U0126 compared to vehicle treatment (Figure 6C). However, total C/EBPβ levels in hESCs were not changed by U0126 treatment (Figure S2). These results suggest that activation of ERK1/2 plays an important role in the decidualization process via phosphorylation of C/EBPβ.

## Discussion

ERK1/2 are proteins well known for regulating proliferation and differentiation. Our results now demonstrate that ERK1/2 plays an important role in decidualization. ERK1/2 is stimulated by EGFR activation, through the GRB2-SOS complex and the Ras-Raf-MEK signaling cascade [29,30]. Phospho-ERK1/2 is first seen on 4.5dpc in the decidualized cells surrounding the embryo in mice (Figure 1). Its expression then expands to include the primary and secondary decidual zones on 5.5 and 7.5 dpc, respectively. Our real-time PCR results show that the following known ERK1/2 target genes, *Fos*, *Stat1, Ubtf, Elk1, Serpine1* and *Msk1* are also significantly up-regulated on 5.5 dpc at the implantation sites. *Fos* is an *ERK1/2* target gene implicated in cell proliferation and differentiation [31]. Our result herein confirm a previous study that *Fos* is expressed in the mouse luminal epithelium and decidual zone during the early implantation period [32]{Baker, 1992 #27}. *Stat1* and *Stat3* expression have been reported in maternal decidual tissues [33]. *Stat3* plays a pivotal role during decidualization [34,35]. However, unlike *Stat3*, decidual *Stat1* appears to be present only in an inactive form. Although its mRNA and protein are abundant [33], its role in decidualization is unclear. *Serpine1*, also known as Plasminogen Activator Inhibitor-1, is a primary inhibitor of fibrinolysis whose expression can be detected in endometrial stromal cells from the luteal phase until (and including) gestation [36,37]. Other ERK1/2 target genes, *Ubtf, Elk1* and *Msk1*, do not yet have established roles in decidualization, but their increased expression patterns in the early pregnant mouse uterus has been well documented. *Msk1* plays a regulatory role in chromatin remodeling in breast cancer cells [38], *Ubtf* is a known target gene for PRA in human endometrial epithelial cell line [39] and *Elk-1* is a member of the ternary complex factor (TCF) subfamily that binds the serum response element found in the promoters of *Fos* and other immediate early genes [40]. *Elk* also upregulates *PTGS2* via the PI3K and ERK1/2 pathways which may be important for decidualization by increasing PGE2 levels [41]. This study found that all of those ERK1/2 target genes are induced at the implantation site during decidualization. These results suggest that activation of ERK1/2 target genes at implantation sites is important for implantation and decidualization.

It is well established that initiation of decidualization is dependent upon progesterone signaling as well as increased levels of cyclic adenosine monophosphate (cAMP), a ubiquitous second messenger molecule generated from ATP by adenylate cyclase [42]. However, this process is complex, occurs at multiple levels, and its mechanism is still largely unknown. There have been multiple studies attempting to identify steroid-regulated pathways related to stromal cell differentiation during embryo implantation [43,44,45]. However, to the best of our knowledge, this is the first study to identify a role for ERK1/2 in decidualization. This study also shows that activation of ERK1/2 phosphorylation is also important for decidualization of hESCs. During *in vitro* decidualization of hESCs, we observed peak induction of ERK1/2 activity on day 3, followed by a tapering in activity on day 6 (Figure 4). These results suggest that phosphorylation of ERK1/2 is required for the induction of decidualization, and then subsequently levels of phospho-ERK1/2 is decreased in stromal cells which have completed the decidualization process. When we treated hESCs with a specific ERK1/2 inhibitor on day1 of artificial decidualization, there was no difference in expression of decidualization marker genes as compared with non-treated hESCs (data not shown). However, when we blocked activation of ERK1/2 on day 3, decidualization of hESCs was reduced as indicated by suppression of *PRL* and *IGFBP1* expression (Figure 5). This result suggests that the timing of ERK1/2 activation is important during endometrial decidualization.

It has been reported that CCAAT/ enhancer binding protein (C/EBPβ) is a critical regulator of endometrial stromal cell proliferation and decidualization [23,24,25,43]. In this study, we found that inhibition of ERK1/2 phosphorylation during *in vitro* decidualization resulted in a concomitant reduction in C/EBPβ phosphorylation (Figure 6). This suggests that C/EBPβ is regulated by ERK1/2 during endometrial decidualization in hESCs. We also found that ERK1/2 target genes (*FOS, MSK1, STAT1*, and *STAT3*) were significantly down-regulated following U0126 (MEK inhibitor) treatment on day 6 of *in vitro* decidualization.

In summary, ERK1/2 is highly expressed during decidualization in both mouse and human. Inhibition of ERK1/2 phosphorylation suppresses decidualization of hESCs as evidenced by decreased expression of the decidual marker genes, *IGFBP1* and *PRL*. This study also found that activation of ERK1/2 during decidualization results in the phosphorylation of C/EBPβ, an important gene previously implicated in the regulation of decidualization [46,47]. These findings have direct implications for our understanding of implantation and may reveal new therapeutic targets for the treatment of endometrial pathologies and infertility.

## Supporting Information

Figure S1 **Localization of total ERK1/2 during in vitro decidualization.** Expression of total ERK1/2 (a, b, c and d) was examined in hESCs during in vitro decidualization at day 0 (a, e, and i), day 1 (b, f and j), day 3 (c, g and k) and day 6 (d, h and l) by immunofluorescence staining. Images (i, j, k and l) were merged with DAPI staining (e, f, g and h).
(PDF)

Figure S2 **Localization of total C/EBPβ during in vitro decidualization after U0126 treatment.** Expression pattern of total C/EBPβ (a, b, c, d) was investigated in hESCs after induction of *in* *vitro* decidualization on day 3 and day 6. hESCs were treated with U0126 from day 3 of decidualization. Images (i, j, k and l) were merged with DAPI staining (e, f, g and h).
(PDF)

Table S1 **Sequences for quantitative Real-time PCR.**
(DOCX)
