# Supplementary figures and images for: Extracellular Signal-Regulated Kinase 1/2 Signaling Pathway Is Required for Endometrial Decidualization in Mice and Human
Source: PLoS One. 2013 Sep 24;8(9):e75282. doi: 10.1371/journal.pone.0075282 (PMC3782496; doi:10.1371/journal.pone.0075282)

Figure S1

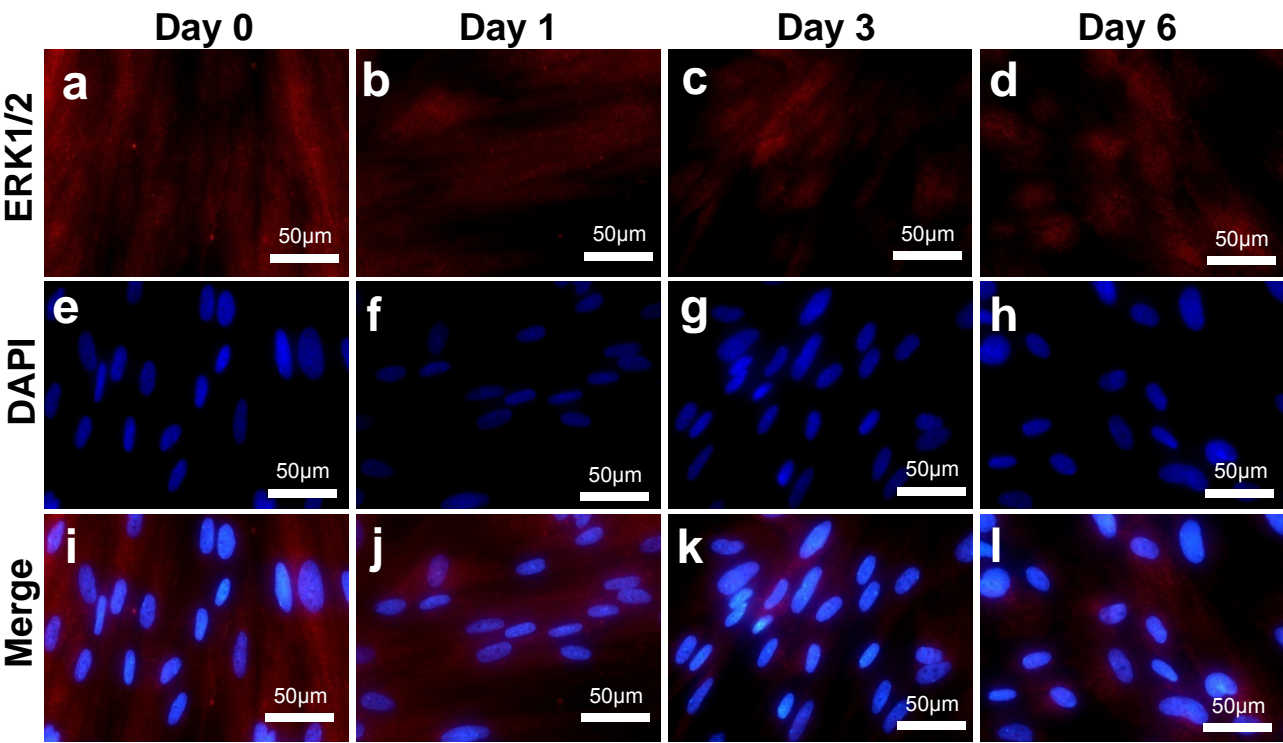

Supplement: Figure S1 — Localization of total ERK1/2 during in vitro decidualization. Expression of total ERK1/2 (a, b, c and d) was examined in hESCs during in vitro decidualization at day 0 (a, e, and i), day 1 (b, f and j), day 3 (c, g and k) and day 6 (d, h and l) by immunofluorescence staining. Images (i, j, k and l) were merged with DAPI staining (e, f, g and h). (PDF) [file pone.0075282.s001.pdf]

Figure S2

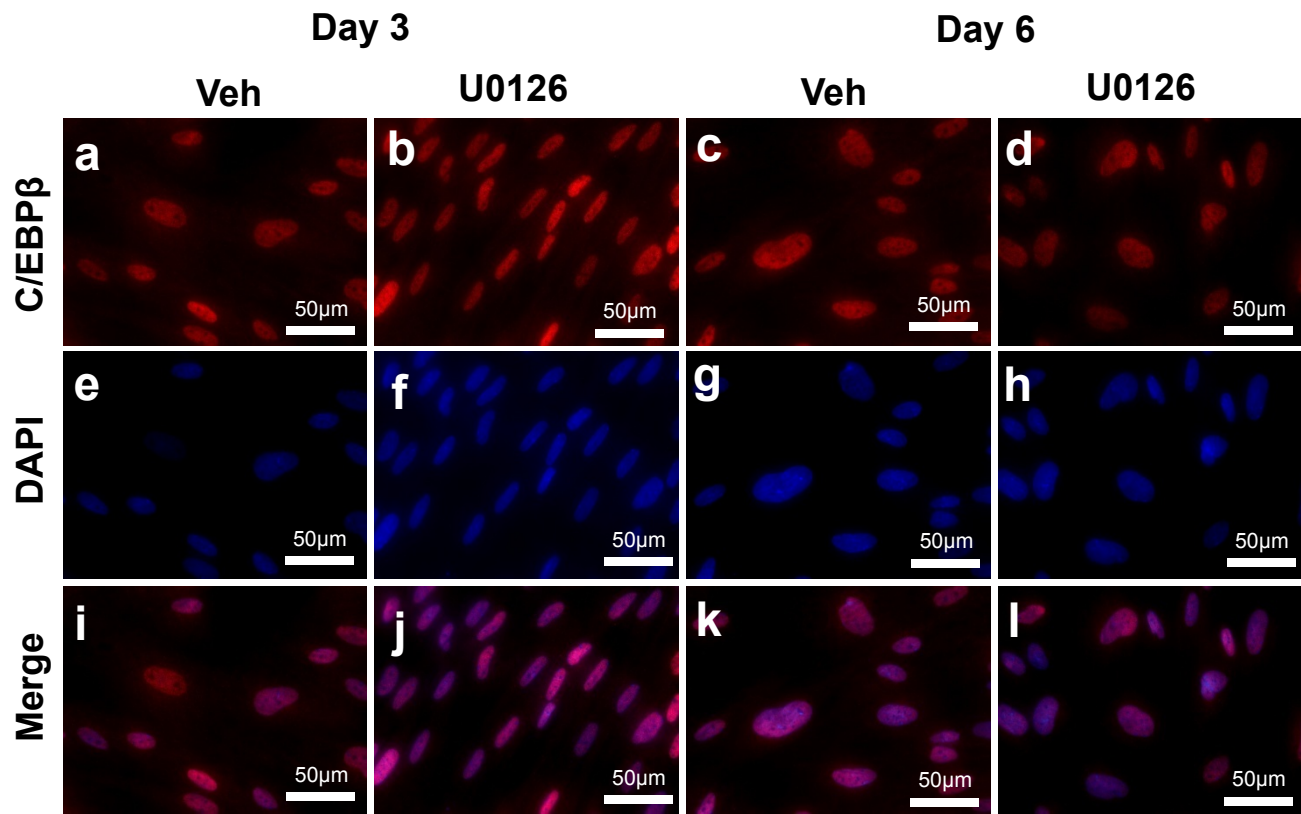

Supplement: Figure S2 — Localization of total C/EBPβ during in vitro decidualization after U0126 treatment. Expression pattern of total C/EBPβ (a, b, c, d) was investigated in hESCs after induction of in vitro decidualization on day 3 and day 6. hESCs were treated with U0126 from day 3 of decidualization. Images (i, j, k and l) were merged with DAPI staining (e, f, g and h). (PDF) [file pone.0075282.s002.pdf]
