# Supplementary material for: Extracellular Signal-Regulated Kinase 1/2 Signaling Pathway Is Required for Endometrial Decidualization in Mice and Human
Source: PLoS One. 2013 Sep 24;8(9):e75282. doi: 10.1371/journal.pone.0075282 (PMC3782496; doi:10.1371/journal.pone.0075282)
Supplement: Table S1 — Sequences for quantitative Real-time PCR. (DOCX) [file pone.0075282.s003.docx]

**Table S1. Sequences for quantitative Real-time PCR.**

| Species | Gene | Sequence | |
| --- | --- | --- | --- |
| Mouse | *18s* | F | GTAACCCGTTGAACCCCATT |
|  |  | R | CCATCCAATCGGTAGTAGCG |
|  | *Fos* | F | CCTTCGGATTCTCCGTTTCTCT |
|  |  | R | TGGTGAAGACCGTGTCAGGA |
|  | *Msk1* | F | GCCGATGAAACTGAAAGAGC |
|  |  | R | TGCTCATTTCCTGGGGATAC |
|  | *Stat1* | F | TGGTGAAATTGCAAGAGCTG |
|  |  | R | TGTGTGCGTACCCAAGATGT |
|  | *Elk1* | F | AGCGGCCAGAAGTTTGTCTA |
|  |  | R | CTGTCATTCCTGCACCCTTT |
|  | *Serpine1* | F | GACACCCTCAGCATGTTCATC |
|  |  | R | AGGGTTGCACTAAACATGTCAG |
|  | *Ubtf* | F | AAGCCATGGAGATGACTTGG |
|  |  | R | GGAGGTTTCTTGGGTTCTCC |
| Human | *18s* | F | GTAACCCGTTGAACCCCATT |
|  |  | R | CCATCCAATCGGTAGTAGCG |
|  | *IGFBP1* | F | CTATGATGGCTCGAAGGCTC |
|  |  | R | TTCTTGTTGCAGTTTGGCAG |
|  | *PRL* | F | CATCAACAGCTGCCACACTT |
|  |  | R | CGTTTGGTTTGCTCCTCAAT |
|  | *FOS* | F | AAGGAGAATCCGAAGGGAAA |
|  |  | R | CTTCTCCTTCAGCAGGTTGG |
|  | *MSK1* | F | CATTAGGCAGTCGCCATTTT |
|  |  | R | GAGGGCAAGCACAATCTCTC |
|  | *STAT1* | F | TGGTGAAATTGCAAGAGCTG |
|  |  | R | AGACTGCCATTGGTGGACTC |
|  | *STAT3* | F | TCACTTGGGTGGAGAAGGAC |
|  |  | R | GCTACCTGGGTCAGCTTCAG |
